# Specific Immune Response and Cytokine Production in CD70 Deficiency

**DOI:** 10.3389/fped.2021.615724

**Published:** 2021-04-30

**Authors:** Hassan Abolhassani

**Affiliations:** ^1^Division of Clinical Immunology, Department of Laboratory Medicine, Karolinska Institutet at Karolinska University Hospital Huddinge, Stockholm, Sweden; ^2^Research Center for Immunodeficiencies, Pediatrics Center of Excellence, Children's Medical Center, Tehran University of Medical Sciences, Tehran, Iran

**Keywords:** primary immunodeficiency, inborn errors of immunity, CD70 deficiency, class-switching recombination, T-bet, Eomes, PD-1

## Abstract

Collective clinical and immunologic findings of defects in the CD27–CD70 axis indicate a primary immunodeficiency associated with terminal B-cell development defect and immune dysregulation leading to autoimmunity, uncontrolled viral infection, and lymphoma. Since the molecular mechanism underlying this entity of primary immunodeficiency has been recently described, more insight regarding the function and profile of immunity is required. Therefore, this study aimed to investigate stimulated antibody production, polyclonal vs. virus-specific T-cell response, and cytokine production of a CD70-deficient patient reported previously with early-onset antibody deficiency suffering from chronic viral infections and B-cell lymphoma. The patient and her family members were subjected to clinical evaluation, immunological assays, and functional analyses. The findings of this study indicate an impaired ability of B cells to produce immunoglobulins, and a poor effector function of T cells was also associated with the severity of clinical phenotype. Reduced proportions of cells expressing the memory marker CD45RO, as well as T-bet and Eomes, were observed in CD70-deficient T cells. The proportion of 2B4^+^ and PD-1^+^ virus-specific CD8^+^ T cells was also reduced in the patient. Although the *CD70*-mutated individuals presented with early-onset clinical manifestations that were well-controlled by using conventional immunological and anticancer chemotherapies, with better prognosis as compared with CD27-deficient patients, targeted treatment toward specific disturbed immune profile may improve the management and even prevent secondary complications.

## Introduction

Common variable immunodeficiency (CVID) is the most common symptomatic primary immune deficiency (PID) characterized by reduced production of immunoglobulins (Igs), predisposing affected individuals to recurrent infections (1, 2). Selected patients may also suffer from autoimmunity, chronic enteropathy, lymphoproliferative disorders, and malignancy (1, 3).

The genetic defects underlying the disease have only been identified in a minority of CVID patients (4, 5). Approximately 5–10% of patients harbor mutations in the *TNFRSF13B* gene (*TACI*) (6). Disease-causing mutations in *TNFRSF13C* (*BAFFR*), encoding one of the ligands for TACI (7); T-cell co-stimulation gene encoding inducible co-stimulator (*ICOS*) (8); B-cell co-receptor complex genes including *CD19, CD81, CD20*, and *CD21* (9–12); genes encoding the lipopolysaccharide-responsive vesicle trafficking beach and anchor containing protein (*LRBA*) (13); cytotoxic T-lymphocyte-associated protein 4 (*CTLA4*) (14); Ras-related C3 botulinum toxin substrate 2 (*RAC2*) (15); phosphatidylinositol 3-kinase (PI3K) receptor 1 (*PIK3R1*) (16); PI3K catalytic subunit delta (*PIK3CD*); phosphatase and tensin homolog (*PTEN*); protein kinase C delta (*PRKCD*) (17); tumor necrosis factor-like weak inducer of apoptosis (*TWEAK/TNFSF12*) (18); nuclear factor-kappa B1 (*NFKB1*) (19); *NFKB2*; and interleukin 21 (*IL21*) (14) and its receptor *IL21R* (20) have also been described in a few patients presenting with features of CVID. Furthermore, mutations in tRNA nucleotidyltransferase 1 (*TRNT1*), IKAROS Family Zinc Finger 1 (*IKZF1*), interferon regulatory factor 2 binding protein 2 (*IRF2BP2*), ATPase proton transporting accessory protein 1 (*ATP6AP1*), Rho guanine nucleotide exchange factor 1 (*ARHGEF1*), SH3 domain-containing kinase-binding protein 1 (*SH3KBP1*), SEC61 translocon Subunit alpha 1 (*SEC61A1*), and mannosyl-oligosaccharide glucosidase (MOGS) have been shown to be associated with the development of the disease (2).

We have recently shown the association of defects in the CD70–CD27 signaling pathway with clinical presentation resembling the CVID phenotype (21–23). CD70 is a co-stimulatory molecule expressed on several types of immune cells including T cells, B cells, and dendritic (DC) cells. Interaction with its ligand, CD27, leads to the signaling cascade in CD70-positive cells *via* anti-apoptotic kinases, in particular PI3K (24–26). On the other hand, CD70 is essential for triggering the survival and proliferation of CD27-positive immune cells through the IL-2-dependent activation of the NFκB pathway (25, 27, 28). Previous animal studies have also implicated the CD70/CD27 pathway in the regulation of immunity vs. tolerance by several mechanisms including T-cell expansion and survival, co-stimulation of antigen presentation, germinal center formation, B-cell activation, and antibody production (29–31). Although several correlations have been observed between deficient patients and animal models, there are still several immune functions and profiling that should be investigated in these newly discovered genetic defects.

## Materials and Methods

### Patients and Immunological Assays

The index family recruited to this study is the first identified family with CD70 deficiency [with confirmed homozygous mutation c.250delT, p.S84Pfs27X (22)]. Written informed consent for this study was obtained from the patients and their relatives, following the principles of the Ethics Committee of Tehran University of Medical Sciences. The immune profile of the index family including complete blood count, lymphocyte subpopulations, serum Ig levels, specific antibody levels, and autoantibodies was documented in our previous report (22, 23, 32). Updated clinical follow-up of the family members was provided, and for more detailed lymphocyte phenotyping, peripheral venous blood was collected both in ethylenediaminetetraacetic acid (EDTA) and later in Streck cell preservative vials (Streck, La Vista, USA) to preserve cells during transport. The samples in the preservative containing vials were processed for flow cytometric immunophenotyping within 36 h. Antibody combinations used for polychromatic 8-color surface staining of lymphocytes have been described previously (33).

### Specific B-Cell Functional Analyses

Peripheral blood mononuclear cells (PBMCs) were also isolated in parallel from whole blood by Hypaque-Ficoll (GE Healthcare) density gradient centrifugation and cryopreserved in freezing medium (Synth-a-Freeze CTS, Life Technologies). Frozen PBMCs were thawed and cultured at 1 × 10^6^ cells per well in 1 ml complete RPMI 1640 (InvivoGen, USA) supplemented with 10% FBS (HyClone Laboratories), either with medium only, or with T-cell-independent stimulation (2.5 μg/ml human CpG oligodeoxynucleotides, InvivoGen + 1 μg/ml Anti-IgM MP Biomedicals, USA) or T-cell-dependent stimulation (10 ng/ml IL-10, R&D, USA + 300 ng/ml CD40L, Immunokemi, Sweden). Cell proliferation was measured on days 0 and 5 using the CellTiter 96 Aqueous One Solution Kit (Promega, USA), following the instructions from the manufacturer. The Ig concentration in the cell culture supernatant was measured at day 7 by standard enzyme-linked immunosorbent assay (ELISA) as described previously (6). Rabbit anti-human IgA and IgM (1:4,000, Dako, UK) and IgG (1:4,000, Jackson ImmunoResearch, USA) were used as capture antibodies, and peroxidase-conjugated goat anti-human IgA, IgG, and IgM were used as secondary antibodies (1:5,000, Dako, UK).

### Specific T-Cell Functional Analyses

The protocol for intracellular staining of transcription factors and functional markers after polyclonal and virus-specific stimulation has been described elsewhere (34). Antibodies used for the functional and phenotypic panels of T cells are shown in Supplementary Table 1. LIVE/DEAD Aqua amine dye (Life Technologies) was used to discriminate dead cells or debris. PBMCs were analyzed on a four-laser LSR Fortessa (BD Biosciences). Antibody capture beads (BD Biosciences) were stained individually with all antibodies used in the experiments for compensation setup. Gating analysis was performed using FlowJo 8.8.7 (TreeStar, Ashland, USA). Manual gates were based on unstained cells or fluorescence minus one (FMO) gating strategies as previously described (34, 35). A T-cell response was considered positive if the frequency of cytokine-producing cells were twice the negative background and >0.1% of total CD4^+^ or CD8^+^ T cells after background reduction.

### Multidimensional Phenotype Clustering Analysis

The manual gating of the flow cytometry data produced CD4^+^ and CD8^+^ T-cell gated data, and the fluorescence shift between the samples was normalized using Gauss norm (36). The visual interactive stochastic neighbor embedding (viSNE) (37) tool was used to map the high-dimensional cytometry data onto a two-dimensional scatterplot. In parallel, the data were analyzed by the PhenoGraph algorithm (38) to allow automated multidimensional clustering of the cells. The subpopulations determined by PhenoGraph were relayed back on to the viSNE map. Heat maps and unsupervised hierarchical clustering of the average fluorescence of each PhenoGraph population were used for visualization. In summary, the flow cytometry data were analyzed using cyt (37), PhenoGraph algorithm (38), SPICE (39), and R environment (40).

## Results

The clinical manifestations [recurrent upper respiratory tract infections, varicella pneumonia, a viral infection of the central nervous system, Behçet's syndrome, alopecia areata, and Epstein–Barr virus (EBV)-mediated Hodgkin lymphoma] of two patients from this index family with shared homozygous mutation of c.250delT, p.S84Pfs27X have been reported (22). At the most recent follow-up, the proband (age 33) was clinically stable on monthly immunoglobulin replacement therapy and prophylactic antimicrobial therapy; she only experienced only one admission during the last 5 years due to pneumonia and no sign of lymphoma relapse was observed; P2 (age 37) was intellectually disabled but did not present with other clinical manifestations and without progression of his humoral immunodeficiency (specific antibody deficiency). Moreover, basic humoral immunologic assays (hypogammaglobulinemia, increased percentage of CD38^+^IgM^+^ transitional B cells and CD27^−^ naive B cells, and a diminished IgM^−^IgD^−^CD27^+^ class-switched memory B cells) have been identified in the previous report of these CD70-deficient patients (22), resembling B-cell phenotype that is most similar to CVID patients who are classified as B^+^smB^−^Tr^hi^ according to the EURO classification (41). Our further investigation on the B-cell subset of the proband indicated a slightly increased frequency of plasmablasts and CD21^low^ B cells (Table 1).

**Table 1:** Extended B-, T-, and NK-cell subsets within the lymphocytes of the proband with homozygous CD70 mutation.

	**Result**	**Reference range**
**B-cell subpopulations**		
CD21^low^CD27^−^ immature B cells (cell/μl)	**23.7↑**	1.1–12.3
CD21^low^CD38^+^ immature transitional B cells (cell/μl)	**47.4↑**	0.4–9.7
CD38^+^IgM^+^ transitional B cells (cell/μl)	**31.2↑**	0.8–8.2
CD38^+^IgM^−^ plasmablasts (cell/μl)	**1.7↑**	0–1.5
CD138^+^ plasma cells (cell/μl)	1.2	0–14.8
CD21^low^CD38^low^ activated B cells (cell/μl)	**42.4↑**	0.5–8
CD20^+^ B cell (% in B cells)	**76.8↓**	86.1–99.4
IgA^+^ B cell (% in B cells)	**1.6↓**	3.4–14.8
**NK-cell subpopulations**		
CD16^−^CD56^−^ immature NK-1 cells (% in NK cells)	**1.1↑**	0–0.5
CD16^−^CD56^+^ immature NK-2 cells (% in NK cells)	9.6	1.7–9.6
CD16^+^CD56^+^ NK-cell mature NK cells (% in NK cells)	56.9	78.1–93.7
CD56^bright^CD16^dim^ regulatory effector NK cells (% in NK cells)	**8.8↑**	1–7
CD94^+^NKG2D^+^CD16^+^CD56^+^ active complex NK cells (% in NK cells)	**20.1↓**	20.4–69
CD3^dim^CD56^−^CD16^+^ cytolytic NK cells (% in NK cells)	**23.9↑**	1–10.1
**T-cell subpopulations**		
CD4^+^CCR7^−^CCR5^+^ Th1 helper T cells (% in helper T cells)	9.2	4.5–25.5
CD4^+^CCR7^−^CCR3^+^ Th2 helper T cells (% in helper T cells)	2.5	1.5–11.3
CD25^+^CD127^low^regulatory T cells (% in Treg cells)	61.4	34.3–98.2
CD25^+^CD127^low^CD45RO^+^ memory Treg (% in Treg cells)	35.2	14–36.9
CD25^+^CD127^low^HLA-DR^+^ activated Treg (% in Treg cells)	18.5	5.9–18.8
CD25^+^CD127^low^CD45RO^−^ naive Treg (% in Treg cells)	19.0	5.6–27.4
CD4^+^CD8^−^CCR6^+^ Th17-like T cells (cell/μl)	**76↓**	119–463
CD45RA^+^CD62L^+^CD45RO^+^ helper T cells (cell/μl)	**0↓**	31.2–62.3
CD45RA^+^CD62L^+^CD45RO^+^ cytotoxic T cells (cell/μl)	**0.2↓**	36.2–69.9
CD45RA^+^CD62L^+^CD31^+^ RTE helper T cells (cell/μl)	324.2	119–487
CD45RA^+^CD62L^+^CD31^+^ RTE cytotoxic T cells (cell/μl)	406.8	64–445
CCR7^+^CD45RA^−^CD62L^+^ CD31^−^ central naive helper T cells (cell/μl)	**0↓**	10.4–26.4
CCR7^+^CD45RA^−^CD62L^+^ CD31^−^ central naive cytotoxic T cells (cell/μl)	**0.1↓**	9.8–25.3
CD4^−^CD8^−^ double negative T cell (% in T cells)	8.8	3–10.2
**NKT-cell subset**		
CD3^+^CD16^+^NKT cells (% in lymphocytes)	7.0	2.1–13.7

*Bolded values indicate out of normal range. ↑ and ↓ depict increased and decreased values compared to healthy individuals reference range, respectively*.

We have also reported increased frequencies of naive CD4^+^ and CD8^+^ T cells, decreases in memory T-cell subsets, and almost normal proportions of NK cells and NK subsets in CD70-deficient patients (22). In line with our previous observation, a more detailed T-cell immunophenotyping was performed for the proband to evaluated T-stem cell memory thymic emigrated (CD45RA^+^CD62L^+^CD45RO^+^) helper and cytotoxic T cells, which showed a reduced number as well as decreased Th17-like T cells. No obvious differences in the proportion of the invariant natural killer T cells (iNKT, TCR Vα24-Jα18) between the proband and normal controls were detected (Table 1).

**Figure 1:**
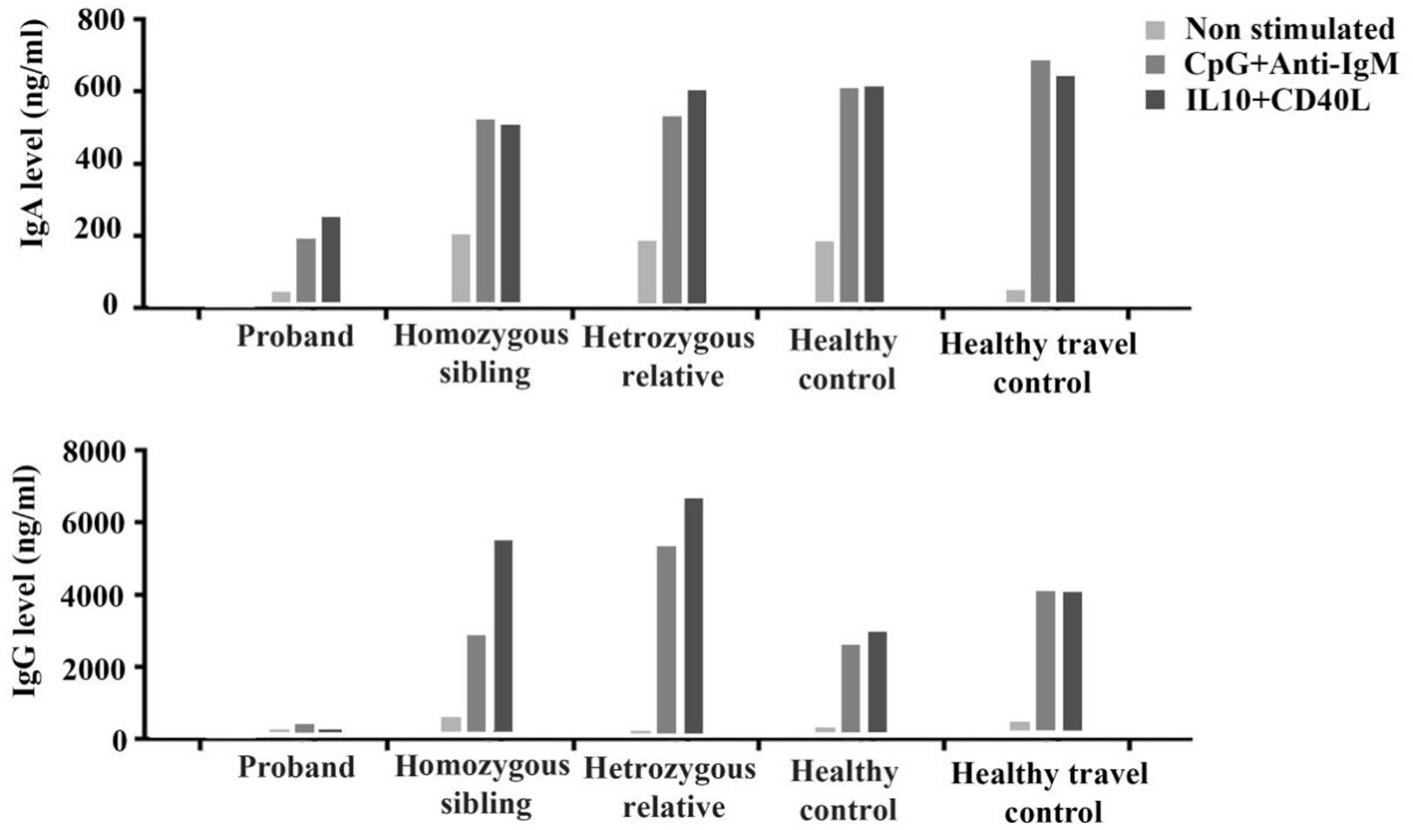
*In vitro* production of IgA and IgG before (non-stimulated) and after stimulation with T-independent (CpG+anti-IgM) and T-dependent (IL-10+CD40L) cytokines in the proband, the homozygous sibling, a heterozygous relative, and two healthy controls.

PBMCs from the proband, the homozygous sibling, a heterozygous relative, a healthy blood donor, and a healthy travel control were tested *in vitro* for their ability to be induced and produce immunoglobulins. The proliferation response to the T-dependent stimuli was normal in both the proband and the homozygous brother (Supplementary Figure 1). Cells from the proband (with severe phenotype), but not the homozygous brother (with milder phenotype), had a reduced ability to respond to T-independent (anti-IgM+CpG) and T-dependent stimuli (IL-10+CD40L) to produce IgA and IgG, especially the latter (Figure 1).

**Figure 2:**
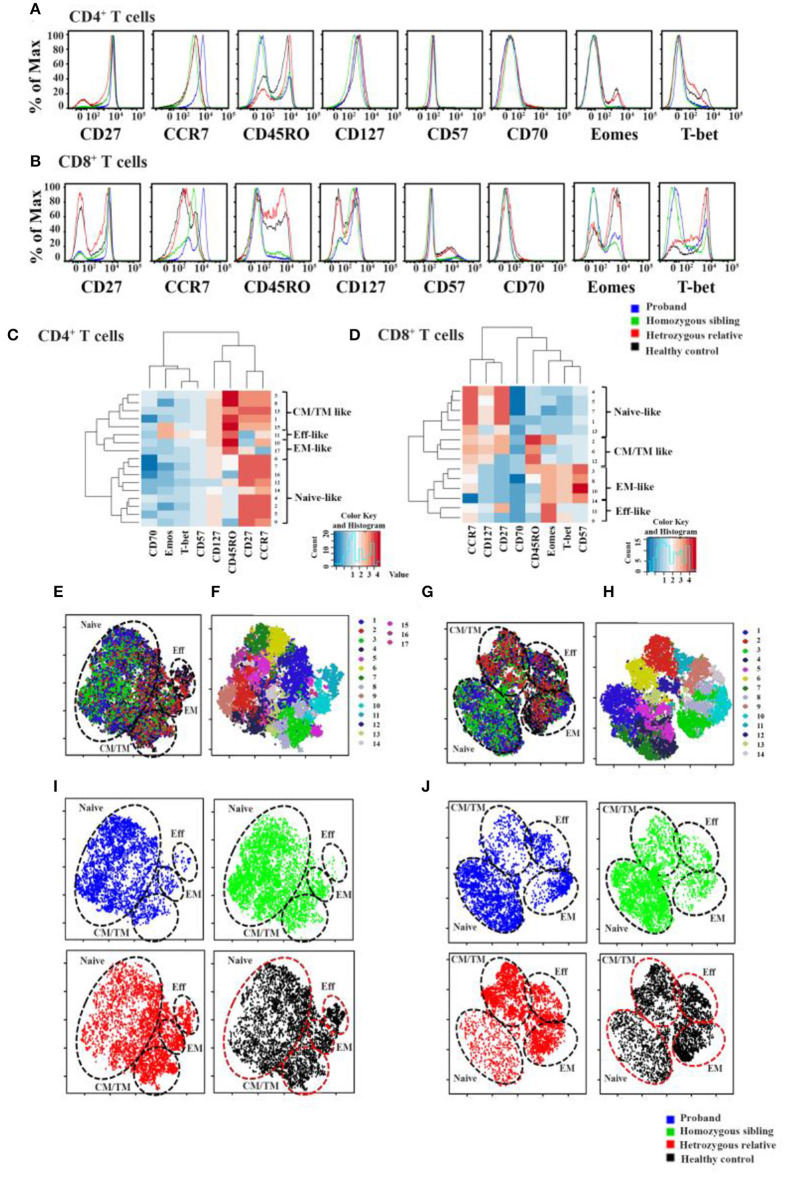
Immunologic markers of cells in CD70-deficient family. Histogram overlays of all differentiation markers (CCR7, CD27, CD57, CD70, CD127, CD45RO, Eomes, and T-bet) for CD4^+^ **(A)** and CD8^+^ cells **(B)** in two homozygous patients, a heterozygous relative father, and a healthy control. Using an automated density-based algorithm called PhenoGraph, 17 CD4^+^ T-cell **(C)** and 14 CD8^+^ T-cell **(D)** subclusters were identified based on the intensity of CCR7, CD27, CD57, CD70, CD127, CD45RO, Eomes, and T-bet expression. Hierarchical Spearman clustering was performed to delineate the relationship between the different T-cell clusters using dendograms and heat maps. The relationships between different clusters were identified as naive-, central/transitional memory- (CM/TM-), effector memory- (EM-), and effector (Eff-) like T-cell populations. Localization of each PhenoGraph-derived subcluster and overlapped plotted viSNE map of individuals within the viSNE map for CD4^+^ and CD8^+^ T cells was shown in **(E,F)** and **(G,H)**, respectively (for more information about each PhenoGraph population, see Supplementary Tables 4, 5). In **(I,J)**, each subject's phenotypic data were plotted on the viSNE map, represented with different colors (blue, proband; green, homozygous brother; red, heterozygous father; black, healthy control). Naive-, CM/TM-, EM-, and Eff-like T-cell populations were marked within the viSNE plot based on the subclusters that were determined using PhenoGraph in sections **(E–H)**.

To further analyze the T-cell profile in CD70-deficient subjects, we developed polychromatic flow cytometry panels to simultaneously distinguish the expression levels of multiple phenotypic markers (CD45RO, CD27, CCR7, CD57, CD127, CD70) and key transcription factors (T-bet and Eomes) on CD4^+^ and CD8^+^ T cells. To achieve a high-dimensional view of the phenotypic and transcriptional heterogeneity of CD4^+^ and CD8^+^ T cells between the subjects, CD4^+^ and CD8^+^ T cells from each subject were analyzed using the PhenoGraph algorithm. PhenoGraph is an automated clustering approach that defines biologically relevant cell populations from multiparametric flow cytometry datasets without any subjective gating. The hierarchical clustering identified CD70 as the main outlier, not clustering well with other markers of T-cell differentiation. Seventeen CD4^+^ T-cell and 14 CD8^+^ T-cell subpopulations were identified based on the combination of the phenotypic and transcriptional flow dataset. By employing hierarchical clustering of the average fluorescence of each PhenoGraph population, we were able to identify T-cell subpopulations that clustered together into naive-, central/transitional memory (CM/TM)-, effector memory (EM)-, and effector (Eff)-like CD4^+^ and CD8^+^ T-cell groups (Figures 2A–D). Detailed expression levels of all T-cell markers for each population are listed in Supplementary Tables 2, 3.

**Figure 3:**
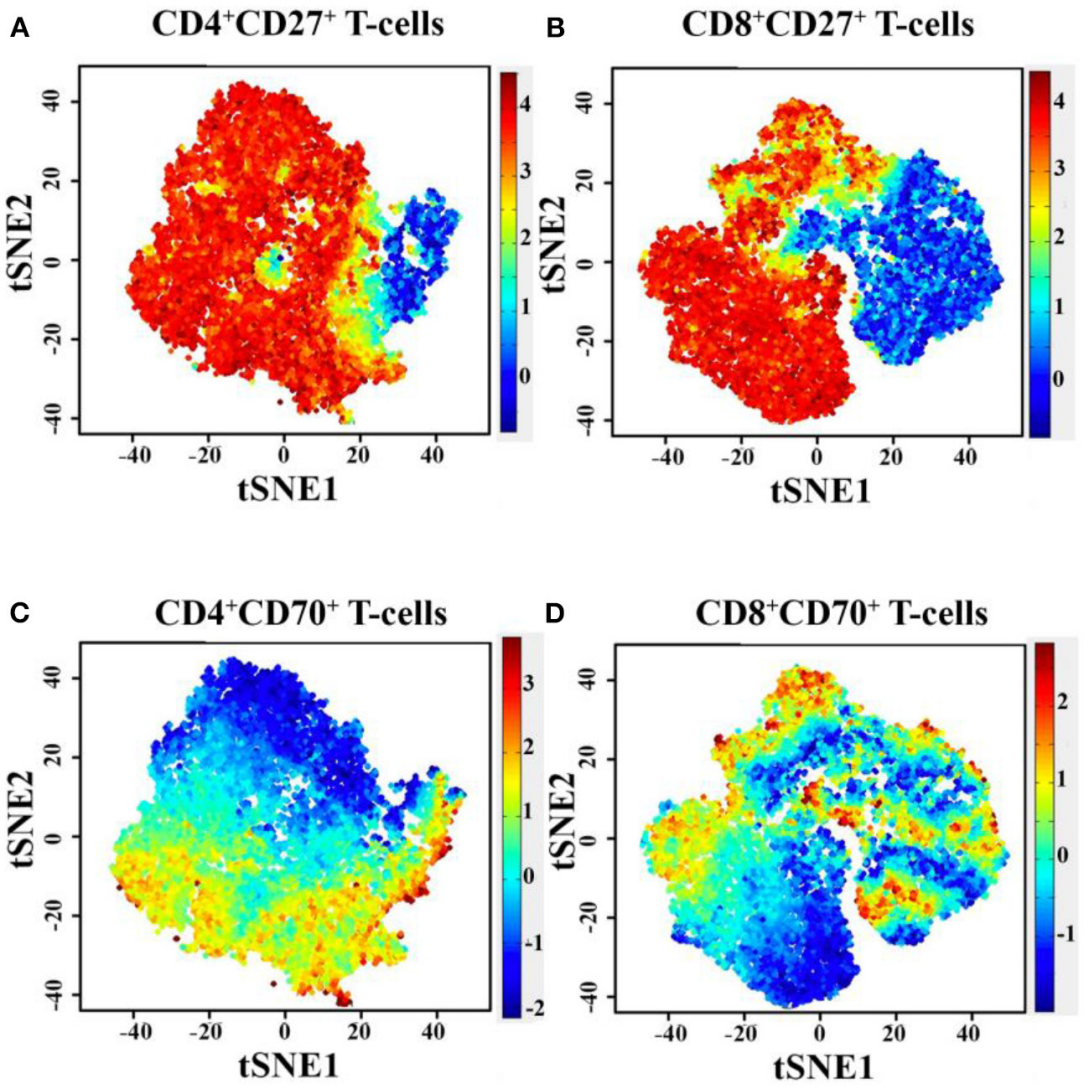
Diagram of PhenoGraph-derived subcluster within the viSNE map, showing the level of CD27 **(A,B)** and CD70 **(C,D)** protein expression on CD4^+^ and CD8^+^ T cells. t-SNE, t-distributed stochastic neighbor embedding.

To generate comparisons of the identified T-cell clusters between the different subjects, we applied the viSNE tool in our analysis (37, 42). This generated a representative map of individual cells similar to biaxial plots, but uses pairwise distances that reflect each cell's proximity in a high-dimensional, rather than a two-dimensional, space (Figures 2E–J). Figure 3 illustrates the distribution of CD27 and CD70-positive CD4^+^ and CD8^+^ T cells on the representative viSNE plot. A general absence of CD70 expression and increased intensity of CD27 in subclusters enriched with cells from the homozygous patients were observed. Notably, reduced proportions of cells expressing the memory marker CD45RO, as well as T-bet and Eomes, were observed in the proband and her homozygous brother, as compared with the heterozygous relative and healthy control. Furthermore, the homozygous patients demonstrated elevated levels of naive and early differentiated T-cell markers (CCR7 and CD127), particularly for CD8^+^ T cells. The viSNE approach demonstrated that approximately 70% of all CD4^+^ T cells for both homozygous individuals were present within different subclusters of naive-like CD4^+^ T cells, equaling twice as many in comparison with the heterozygous relative and the healthy control. Conversely, fewer cells were present in different EM- and Eff-like clusters in both homozygous patients, especially for the Eff-like cluster 11 expressing both T-bet and Eomes (Figure 2E and Supplementary Table 2). In addition, both homozygous patients showed a general lack of CD45RO^+^ cells and possessed 3–6 times higher frequencies of naive-like CD8^+^ T cells than the heterozygous relative and the control. Although some variation was noted between the two homozygous patients, they possessed low frequencies of mainly EM-like (such as clusters 3 and 8) or Eff-like cells co-expressing both T-bet and Eomes (cluster 9) (Figure 2G and Supplementary Table 3).

**Figure 4:**
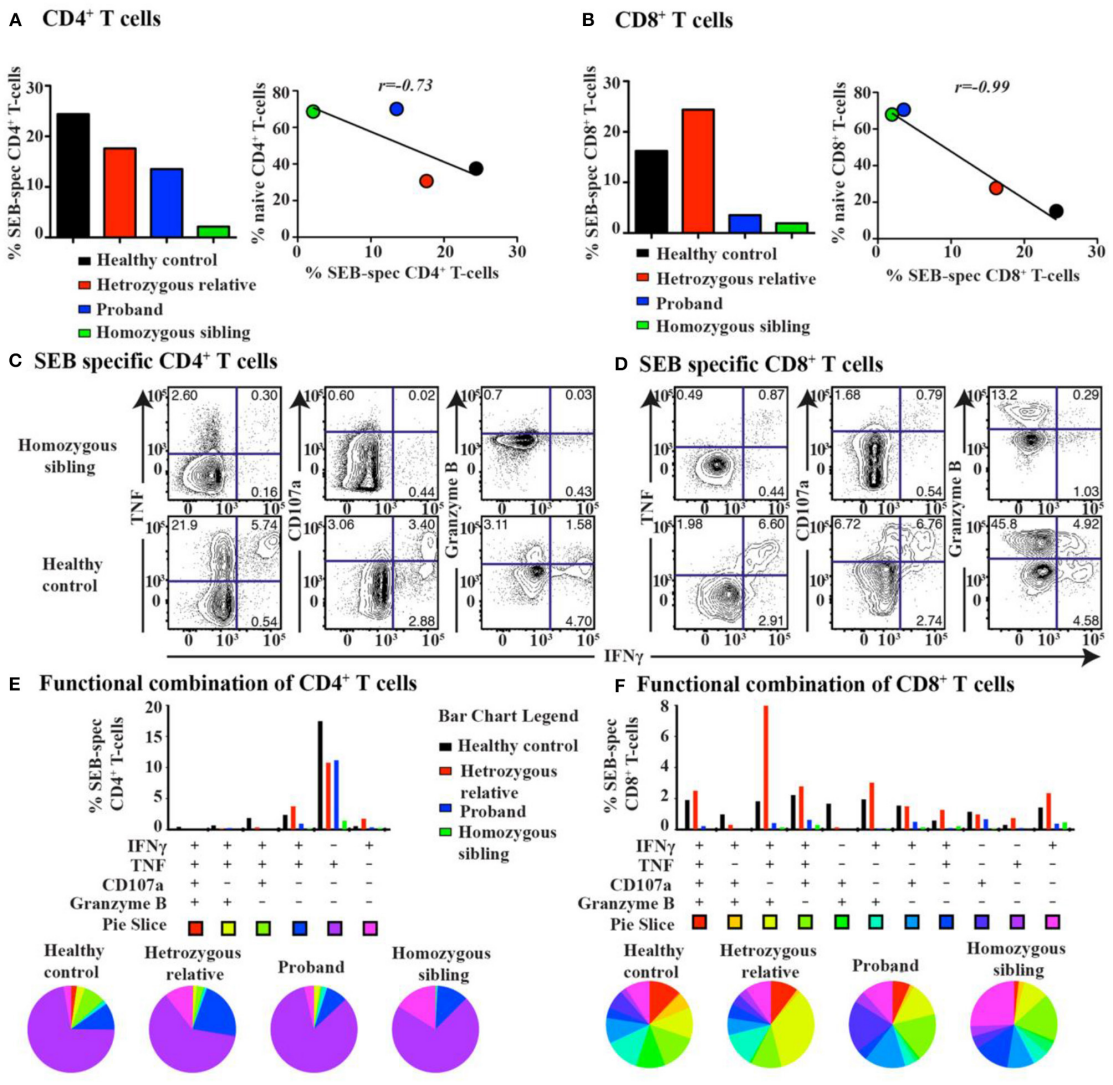
T-cell response and cytokine production after super-antigen simulation in CD70 deficiency. The total frequency of super-antigen (SEB)-specific **(A)** CD4^+^ and **(B)** CD8^+^ T cells (left). Pearson correlation analysis between the frequency of SEB-specific T cells and naive T cells for all subjects (right). Representative biaxial plots of SEB-specific **(C)** CD4^+^ and **(D)** CD8^+^ T-cell expression of IFNγ together with TNF, CD107a, or granzyme B for a homozygous patient and a healthy control. Corresponding SPICE analysis of all functional combinations (based on Boolean gating) for each patient's SEB-specific **(E)** CD4^+^ and **(F)** CD8^+^ T-cell response (below). Functional combinations with no detectable response (<0.05%) are not depicted in the graph.

**Figure 5:**
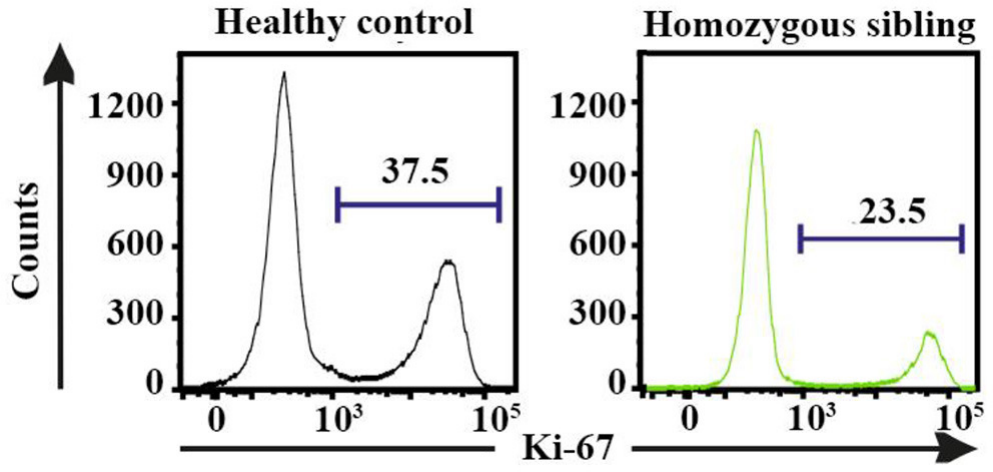
The proliferative (Ki-67^+^) response for staphylococcal enterotoxin B-specific CD8^+^ T cells following 3-day stimulations in the homozygous patient and a healthy control.

Based on the multidimensional phenotypic and transcriptional T-cell profiling data, we next sought to determine the functional characteristics of T cells in CD70-deficient patients. Polyclonal stimulation using Staphylococcal enterotoxin B (SEB) further confirmed that particularly CD8^+^, but also CD4^+^, T cells contained low frequencies of responding cells. The lower proportion of SEB-polyclonal CD8^+^ T cells was strongly associated with a higher frequency of naive T cells in both homozygous individuals (Figures 4A,B). The expression pattern of several effector molecules (IFNγ, TNF, CD107a, and granzyme B) for polyclonal (SEB)-specific T cells was subsequently assessed (Figures 4C,D and Supplementary Tables 4, 5). The SEB stimulations confirmed that particularly CD8^+^, but also CD4^+^, T cells possessed low frequencies of responding cells (Figures 4C,D). By employing SPICE analysis to determine the co-expression pattern of all functional markers (Figures 4E,F), we observed that for both of the homozygous patients, there was a clear lack of cells producing two or more cytokines, indicating that Th1 and cytolytic CD4^+^ T-cell polarization is severely affected by the lack of CD70 (Figure 4E and Supplementary Table 4), as previously demonstrated in murine models (43). In line with the CD4^+^ T-cell data, polyclonal stimulations revealed that both homozygous cases had very low frequencies of CD8^+^ T cells capable of producing multiple cytokines after polyclonal stimulations (Figure 4F and Supplementary Table 5). The proliferative response (Ki-67 expression) for a healthy control and proband was also assessed following a 3-day stimulation with the polyclonal antigen SEB. The homozygous proband possessed fewer CD8^+^ T cells (23.5%) that proliferated in response against SEB than the healthy subject (37.5%, Figure 5).

**Figure 6:**
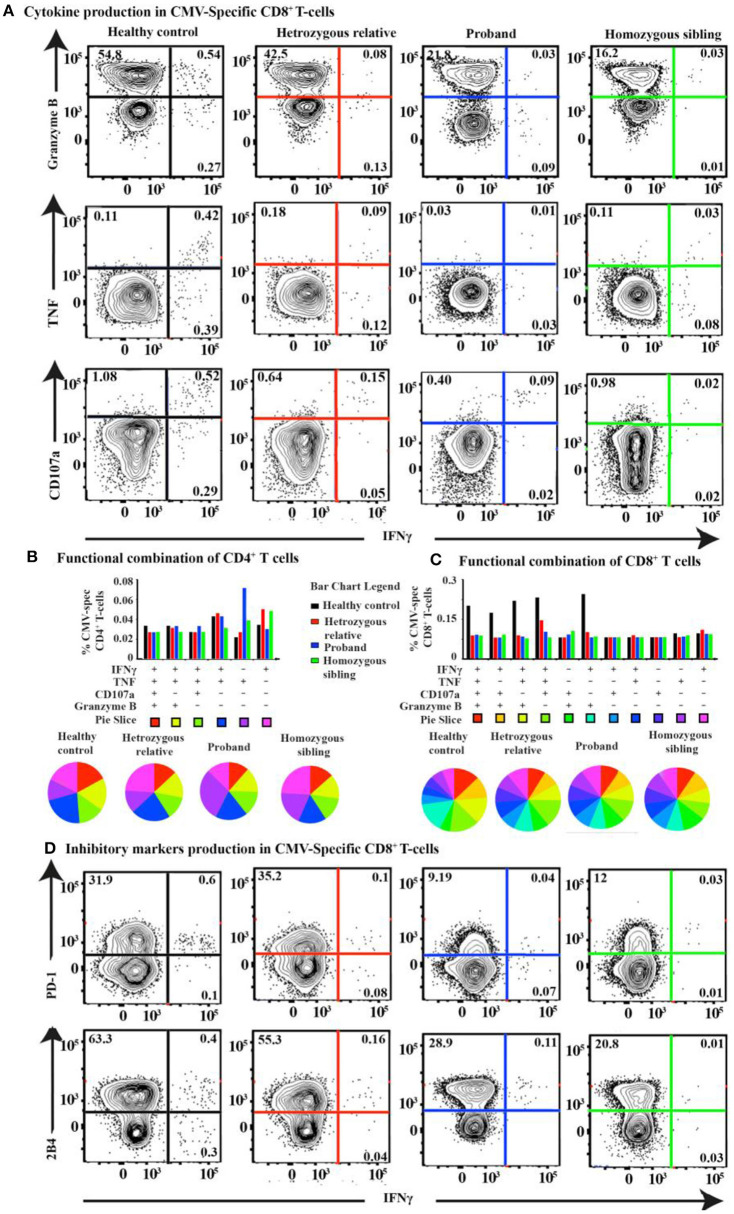
T-cell response and cytokine production after CMV-pp65 antigen simulation in CD70 deficiency. **(A)** FACS plots of the IFNγ vs. granzyme B, TNF, and CD107a CMV-pp65-specific CD8^+^ T-cell response for the proband, the homozygous sibling, a heterozygous relative, and a healthy control. Corresponding SPICE analysis of all functional combinations (based on Boolean gating) for each patient's CMV-specific **(B)** CD4^+^ and **(C)** CD8^+^ T-cell response. **(D)** FACS plots of the IFNγ vs. PD-1 and 2B4 CMV-pp65-specific CD8^+^ T-cell responses.

In our previous report, we presented impaired specific immunity against Epstein–Barr virus (EBV) aligned with high viral and antibody titers for EBV. However, we also observed increased antibody titers against cytomegalovirus (CMV), herpes simplex virus 1 (HSV-1), and varicella-zoster virus (VZV) in the homozygous patients (22), suggesting CD70 deficiency is associated with increased susceptibility to infections by the *Herpesviridae* family. We, therefore, extended our functional T-cell analyses to assess the CMV-specific T-cell response. A barely detectable response against HCMV-pp65 was observed both in the homozygous patients and heterozygous individuals. The healthy control, however, clearly showed a positive cytolytic response against HCMV-pp65 (Figures 6A–C and Supplementary Tables 4, 5). Intriguingly, the proportion of 2B4^+^ and PD-1^+^ CMV-specific CD8^+^ T cells was reduced in the proband and the homozygous sibling compared with a heterozygous relative and a healthy control after HCMV-pp65 stimulation (Figure 6D).

## Discussion

In our previous study, we have shown that the clinical and immunologic findings on the first two CD70-deficient individuals indicate a terminal B-cell developmental defect and a reduced T-cell effector function underlying their antibody deficiency, susceptibility to viral infection, autoimmunity, and lymphoid malignancy. CD27–CD70 interaction is not essential for B-cell development but has been suggested to be important for B-cell activation, germinal center (GC) formation, generation of plasma cells, and Ig production (24, 25, 44). This was in line with the current observations in our CD70-deficient proband that besides hypogammaglobulinemia and reduced proportion of switched memory B cells, the patient had an impaired ability of B cells to secrete IgG and IgA *in vitro*. In CD27-deficient mice, the levels of virus-specific Igs in the serum and the frequency and pattern of somatic hypermutation (SHM) of GC B cells in response to conjugated chicken γ globulin seem to be normal (45). In a CD70-deficient mouse model, only T-cell function has been thoroughly studied (43, 46). Nevertheless, concerning B-cell function, these mouse models may not fully reflect the role of CD27–CD70 in humans, as CD27 is expressed on a much higher frequency of human GC B cells and may have a more profound impact on GC reactions such as class-switching recombination and SHM in human cells (25). In support of this view, antibody deficiency is also one of the main clinical features in CD27-deficient patients (21, 23). One question remains, however, if the hypogammaglobulinemia is primarily due to intrinsic B- and T-cell defects and/or whether it is secondary to EBV or other viral infections. In several CD27-deficient patients described previously, the serum level of Igs was normal/high initially but subsequently declined dramatically in the months following documented EBV infections (47, 48). The mechanism linking hypogammaglobulinemia and viral infections, however, remains elusive.

Of note, almost 25% of the B cells did not express CD20 which was associated with <2% frequency of plasma cells/plasmablast and the remission phase of lymphoma and absence of treatment with anti-CD20 monoclonal antibodies. Since no mutation was observed in negative or positive regulators of CD20 (data not shown), defective CD27/CD70 signaling might indirectly impact the CD20 regulators, a notion which has been suggested by previous studies (25, 49–51). Moreover, reflecting the discrepancy in the humoral immunity in these homozygous siblings (one with CVID phenotype and another with specific antibody deficiency), the *in vitro* study showed the impaired ability of B cells to secrete IgG and IgA in proband but not in the homozygous brother. Intriguingly, the high percentage of CD21^low^CD38^low^ B-cell subset (10% of total B cells) might be the reason underlying low B-cell response and also probably the specific autoimmune response in the proband but not the homozygous brother, a notion which was supported by the normal count of this specific subset in the latter patient (CD21^low^CD38^low^ B cell: 2.4%).

We have identified in our previous report that CD27–CD70 co-stimulation is essential for T-cell activation and CD8^+^ memory effector functions as illustrated by *in vitro* analyses (25) and data derived from CD70 knockout mouse models (43, 46, 52). Investigations in our patients provided evidence for a reduced proliferative capacity and cytokine production in both CD4^+^ and CD8^+^ T cells in response to either polyclonal stimulation or CMV-specific antigens. Furthermore, the patients' T cells presented with a more “naive”-like phenotype, with reduced expression of the memory T-cell marker (CD45RO) as well as the transcription factors T-bet and Eomes. These new findings support our previous observation on EBV-specific CD8^+^ T cells from the patients that exhibited a naive-like phenotype (22). Thus, although the T-cell number and subsets were largely unaffected, the T-cell effector function and differentiation of naive precursors into memory cells were abnormal in CD70-deficient patients. As we have shown in our previous study that stem cell-like memory (CD45RA^+^CCR7^+^CD95^−^) has been decreased in association with increased naive markers on CD4 and CD8 T cells, our new observation supports that stem cell-like memory emigrant T cells (CD45RA^+^CD62L^+^CD45RO^+^) are reduced, but total recent thymic emigrant T-cell (CD45RA^+^CD62L^+^CD31^+^) subsets were normal both in helper and cytotoxic T cells in the proband with CD70 deficiency. Considering the recurrent viral (particularly EBV) infection in the proband and distinct functional role of stem cell-like memory compared to other naive emigrant T cells (despite shared similar recirculation patterns and distribution), the CD70–CD27 axis is critical for the development of the former subset with minimally differentiated T cells (53).

Moreover, although NK-cell effector function was normal in CD70^−/−^ mice (46), a reduction of several activation-regulating markers on NK cells was observed in the CD70-deficient proband. In this context, failure to trigger CD27 on NK cells through CD70 directly affects NK activity by decreasing effector–target conjugate formation and IFNγ production of freshly isolated CD27^hi^CD56^bright^ NK cells (54, 55). The spectrum of viral infections seen in CD70-deficient patients is reminiscent of those in other patients with NK-cell deficiencies (e.g., *GATA2* and *FCGR3A* mutations) presenting with susceptibility to *Herpesviridae* virus infection due to defect in targeted NK-cell-mediated defenses by downmodulating major histocompatibility complex (MHC) class 1 (56). Taken together, reduced T-cell function and a subtle defect in NK cells may thus underlie the susceptibility to *Herpesviridae* virus infection in these patients. This also suggests that although several other T-cell co-stimulation pathways exist, such as the OX40/OX40L (57) and 4-1BB/4-1BB-L pathways, the CD27/CD70 interaction has a unique functional role in fine-tuning T-cell-mediated immunity to virus infections.

Behçet's syndrome and alopecia areata were diagnosed in the proband, thereby providing a potential link between CD70 deficiency and a higher risk for autoimmunity. Behçet's syndrome is a rare, systemic inflammatory vascular disorder with unknown etiology, although associations with HLA-B^*^51 and several non-MHC loci including *IL23R-IL12RB2* and *IL-10* have been described (58). Besides, environmental factors such as infections, especially viral infections, have been considered to trigger the disease in genetically predisposed individuals (59). Notably, although not diagnosed as Behçet's syndrome, several CD27-deficient patients also suffer from uveitis and EBV-induced oral/perianal ulcers, which are typical signs of this syndrome (21). Furthermore, there have also been some indications that the onset of alopecia areata is associated with EBV infection (60). Our data would therefore suggest that uncontrolled or chronic viral infections, especially with EBV, due to lack of CD27–CD70 co-stimulation, trigger the autoimmunity observed in the index patient. The CD27–CD70 axis has also been suggested to play a role in central tolerance and the development of regulatory T cells (Tregs) (61, 62) and Th17 cells (63). Moreover, overexpression of CD27 and its soluble form and surprisingly also a higher expression of CD70 have been documented in several types of autoimmune disorders including systemic lupus erythematosus, rheumatoid arthritis, multiple sclerosis, and psoriasis (64, 65). Thus, additional mechanisms may explain the phenotype of our patient, and dysregulation of the CD27–CD70 pathway can contribute to various forms of inflammatory and autoimmune disorders, although additional investigations are required to fully understand the mechanisms involved (66).

The lymphoid malignancy observed in the CD70-deficient proband and various other cancers in the family members suggest that CD70 has a potential role in preventing tumorigenesis. There are two major, mutually nonexclusive hypotheses (67): [1] CD27–CD70 interaction is important for generating NK- and T-cell-mediated antivirus responses; thus, the CD70 mutations cause a defective response to the *Herpesviridae* family, leading to lymphoid proliferation and malignancy; and [2] CD27–CD70 interaction is important for generating NK- and T-cell-mediated antitumor responses, and thus, mutations in CD70 could be considered as a mechanism of immune evasion where malignant cells escape circumvent NK- and T-cell surveillance. In line with these notions, somatic mutations and deletions affecting both alleles of *CD70* have been observed in diffuse large B-cell lymphomas (DLBCLs), supporting its potential role as a tumor suppressor (68, 69). However, paradoxically, a higher expression of CD70 in DLBCL is associated with an unfavorable prognosis, and constitutive expression of CD70 has also been described in other types of lymphomas including Hodgkin's lymphoma and mantle cell lymphoma, as well as solid tumors such as clear cell, ovarian, and nasopharyngeal carcinomas (70). Thus, several oncogenic roles of CD70 have been proposed, including recruitment of CD27^+^ Treg, induction of apoptosis of lymphocytes, and skewing toward T-cell exhaustion (71–73). Therapies targeting the CD27/CD70 axis have furthermore been shown to have some, though not dramatic, clinical effects in kidney cancer patients (70). Our patient lacked CD70 expression, had an EBV^+^ lymphoma, and showed impaired CD8^+^ T-cell memory and effector functions. Furthermore, the heterozygous family members presented with various types of cancers. Thus, our result would argue for a tumor suppressor role for CD70, although further analyses would be required to dissect the function of the CD27–CD70 axis in tumorigenesis.

In conclusion, we have extended our previous observation of the first cases of CD70 deficiency in humans (Table 2). Based on the current study, although CD70 appears not to be essential for T- and B-cell development, together with its ligand CD27, it plays a unique role in T-cell-mediated (T-cell activation and CD8^+^ memory effector function) and B-cell-mediated (B-cell activation, GC formation, generation of plasma cells) immunity. Impaired B-cell Ig induction, poor effector, and memory function of T cells with reduced proportions of CD45RO, T-bet, and Eomes were documented in these patients and need to be evaluated in the future on other patients reported with this group of primary immunodeficiency.

**Table 2:** A summary of new findings of patients with CD70 deficiency.

**Key findings**
• Reduced proportions of cells expressing the memory marker CD45RO, as well as T-bet and Eomes, were observed in CD70-deficient T cells.
• The proportion of 2B4^+^ and PD-1^+^ virus-specific CD8^+^ T cells was reduced in the patients.
• Th17 cells and central naive T cells were significantly reduced in CD70-deficient proband.
Despite increased naive markers on CD4 and CD8 T cells, stem cell-like memory emigrant T cells (CD45RA+CD62L+CD45RO+) were reduced, but the total recent thymic emigrant T-cell (CD45RA+CD62L+CD31+) subset was normal both in helper and cytotoxic T cells.
• Almost 25% of the CD19+ B cells did not express CD20 markers in the proband with severe clinical presentation.
• *In vitro* impaired ability of B cells to produce immunoglobulins and a poor effector function of T cells was associated with the severity of clinical phenotype in patients.
• Expansion of CD21–CD38– subset could be a reason underlying low B-cell response and also probably the specific autoimmune response in the proband but not the homozygous brother.

## Data Availability Statement

The raw data supporting the conclusions of this article will be made available by the authors, without undue reservation, to any qualified researcher.

## Ethics Statement

The studies involving human participants were reviewed and approved by the Regional Ethical Review Board (EPN) in Tehran University of Medical Sciences, Tehran, Iran. Written informed consent to participate in this study was provided by the participants.

## Author Contributions

HA performed the conception and design of the study, acquisition of data, analysis and interpretation of data, drafting the article, and has approved it for publication.

## Conflict of Interest

The author declares that the research was conducted in the absence of any commercial or financial relationships that could be construed as a potential conflict of interest.
