# Supplementary material for: Specific Immune Response and Cytokine Production in CD70 Deficiency
Source: Front Pediatr. 2021 Apr 30;9:615724. doi: 10.3389/fped.2021.615724 (PMC8120026; doi:10.3389/fped.2021.615724)
Supplement: Supplementary file 1 [file Data_Sheet_1.pdf]

**Supplementary data**

**Frontiers Pediatrics/Research Topic:**

**Meaningful Cases of Primary Immunodeficiencies, Volume III.**

# Specific Immune Response and Cytokine Production in CD70 deficiency

Hassan Abolhassani

**Table S1.** The antibodies were used for the functional and phenotypic panels of B- and T-cells.

| No. | Antibody | Fluorochrome | Clone | Company |
|---|---|---|---|---|
| 1 | anti-CD3 | APC-H7 | Clone SK7 | BD Bioscience |
| 2 | anti-CD14 | V500 | Clone M5E2 | BD Bioscience |
| 3 | anti-CD19 | V500 | Clone B43 | BD Bioscience |
| 4 | anti-CD19 | BV711 | Clone SJ25C1 | BD Bioscience |
| 5 | anti-CCR7 | PE-Cy7 | Clone 3D12 | BD Bioscience |
| 6 | anti-CD160 | AF488 | Clone BY55 | BD Bioscience |
| 7 | anti-IFNγ | AF700 | Clone B27 | BD Bioscience |
| 8 | anti-TNF | PE-Cy7 | Clone MAb11 | BD Bioscience |
| 9 | anti-CD107a | PE-CF594 | Clone H4A3 | BD Bioscience |
| 10 | anti-Ki-67 | FITC | Clone B56 | BD Bioscience |
| 11 | anti-2B4 | PE-Cy5 | Clone C1.7 | Beckman Coulter |
| 12 | anti-CD45RO | ECD | Clone UCHL1 | Beckman Coulter |
| 13 | anti-CD57 | FITC | Clone NC1 | Beckman Coulter |
| 14 | anti-T-bet | BV605 | Clone B10 | Biolegend |
| 15 | anti-CD4 | BV785 | Clone OKT4 | Biolegend |
| 16 | anti-PD-1 | BV421 | Clone EH12.2H7 | Biolegend |
| 17 | anti-CD27 | BV785 | Clone O323 | Biolegend |
| 18 | anti-IL-2 | BV605 | Clone MQ1-17H12 | Biolegend |
| 19 | anti-CD70 | PE | Clone 113-16 | Biolegend |
| 20 | anti-CD127 | BV421 | Clone A019D5 | Biolegend |
| 21 | anti-T-bet | PE | Clone B10 | eBioscience |
| 22 | anti-Eomes | EF660 | Clone WD1928 | eBioscience |
| 23 | anti-CD8 | Qd565 | Clone 3B5 | Life Technologies |
| 24 | anti-CD4 | PE-Cy5.5 | Clone S3.5 | Life Technologies |
| 25 | anti-Granzyme B | PE-Cy5.5 | Clone GB11 | Life Technologies |
| 26 | anti-CD57 | FITC | Clone NK-1 | BD Pharmingen |
| 27 | anti-2B4 | PE | Clone eBioC1.7 | eBioscience |
| 28 | anti-PD1 | Biotin | Clone eBioJ105 | eBioscience |
| 29 | anti-CD160 | PE | Clone BY55 | BD Pharmingen |
| 30 | anti-NKG2D | PerCP-eFluor 710 | Clone 1D11 | eBioscience |

**Table S2.** Number of cell from subsampled 5000 cells used for multidimensional phenotype clustering analysis among 17 PhenoGraph populations of CD4$^{+}$ T-cells regarding different clusters of Naïve-, Central/Transitional memory- (CM/TM-), Effector memory- (EM-), and Effector (Eff-) like T-cells in two CD70 deficient homozygous patients, a heterozygous relative and a healthy control.

| Population | Phenotype | Characteristic surface markers | Proband | Homozygous sibling | Heterozygous relative | Healthy control |
|---|---|---|---|---|---|---|
| 3 | CM/TM | CD45RO$^{+}$CCR7$^{+}$CD27$^{+}$ Eomes$^{low}$CD127$^{low}$ CD70$^{low}$ | 339 | 304 | 595 | 441 |
| 8 | CM/TM | CD45RO$^{+}$CCR7$^{+}$CD27$^{+}$ Eomes$^{-}$CD127$^{+}$ CD70$^{low}$ | 216 | 242 | 490 | 372 |
| 13 | CM/TM | CD45RO$^{+}$CCR7$^{+}$CD27$^{hi}$ Eomes$^{low}$CD127$^{+}$ CD70$^{low}$ | 133 | 132 | 170 | 157 |
| 1 | CM/TM | CD45RO$^{+}$CCR7$^{+}$CD27$^{+}$ Eomes$^{low}$CD127$^{+}$CD70$^{-}$ | 487 | 330 | 961 | 849 |
| 15 | CM/TM | CD45RO$^{+}$CCR7$^{+}$CD27$^{+}$Eomes$^{+}$ CD127$^{+}$ CD70$^{low}$ | 43 | 44 | 303 | 96 |
| 11 | Eff | CD45RO$^{+}$CCR7$^{-}$CD27$^{-}$Eomes$^{+}$ T-bet$^{+}$CD57$^{+}$CD127$^{+}$ | 73 | 19 | 370 | 581 |
| 10 | EM | CD45RO$^{+}$CCR7$^{-}$CD45RO+CCR7$^{-}$CD27$^{+}$ CD127$^{+}$ | 107 | 210 | 483 | 427 |
| 17 | EM | CD45RO$^{+}$CCR7$^{-}$CD27$^{-}$CD127$^{low}$ | 17 | 46 | 28 | 50 |
| 6 | Naïve | CD45RO$^{low}$ CCR7$^{+}$CD27$^{hi}$Eomes$^{low}$T-bet$^{low}$ CD127$^{+}$CD70$^{-}$CD57$^{low}$ | 510 | 296 | 248 | 345 |
| 7 | Naïve | CD45RO$^{-}$CCR7$^{+}$CD27$^{hi}$ Eomes$^{-}$T-bet$^{-}$ CD127$^{+}$CD70$^{-}$CD57$^{low}$ | 553 | 331 | 222 | 278 |
| 16 | Naïve | CD45RO$^{-}$CCR7$^{+}$CD27$^{hi}$ Eomes$^{-}$T-bet$^{low}$ CD127$^{+}$CD70$^{-}$CD57$^{low}$ | 125 | 171 | 71 | 83 |
| 12 | Naïve | CD45RO$^{-}$CCR7$^{+}$CD27$^{hi}$ Eomes$^{-}$T-bet$^{-}$ CD127$^{-}$CD70$^{-}$CD57$^{-}$ | 194 | 410 | 101 | 208 |
| 14 | Naïve | CD45RO$^{-}$CCR7$^{+}$CD27$^{+}$ Eomes$^{-}$T-bet$^{low}$ CD127$^{low}$CD70$^{-}$CD57$^{low}$ | 58 | 305 | 143 | 49 |
| 4 | Naïve | CD45RO$^{-}$CCR7$^{+}$CD27$^{hi}$Eomes$^{low}$T-bet$^{low}$CD127$^{+}$CD70$^{low}$CD57$^{low}$ | 559 | 434 | 197 | 331 |
| 2 | Naïve | CD45RO$^{-}$CCR7$^{+}$CD27$^{hi}$ Eomes$^{-}$T-bet$^{-}$CD70$^{low}$CD57$^{low}$ | 716 | 706 | 256 | 294 |
| 5 | Naïve | CD45RO$^{-}$CCR7$^{+}$CD27$^{hi}$Eomes$^{low}$T-bet$^{low}$ CD127$^{+}$CD70$^{-}$CD57$^{low}$ | 525 | 421 | 215 | 280 |
| 9 | Naïve | CD45RO$^{-}$CCR7$^{+}$CD27$^{hi}$Eomes$^{-}$T-bet$^{low}$ CD127$^{+}$CD70$^{low}$CD57$^{low}$ | 345 | 599 | 147 | 159 |

**Table S3.** Number of cell from subsampled 5000 cells used for multidimensional phenotype clustering analysis among 14 PhenoGraph populations of $CD8^{+}$ T-cells regarding different clusters of Naïve-, Central/Transitional memory- (CM/TM-), Effector memory- (EM-), and Effector (Eff-) like T-cells in two CD70 deficient homozygous patients, a heterozygous relative and a healthy control.

| Population | Phenotype | Characteristic surface markers | Proband | Homozygous sibling | Heterozygous relative | Healthy control |
|---|---|---|---|---|---|---|
| 4 | Naïve | $CD45RO^{-}CCR7^{+}CD27^{hi}$ $Eomes^{low}T\text{-}bet^{-}$ $CD127^{+}CD70^{-}CD57^{low}$ | 936 | 472 | 121 | 305 |
| 5 | Naïve | $CD45RO^{-}CCR7^{+}CD27^{hi}$ $Eomes^{+}T\text{-}bet^{-}$ $CD127^{low}CD70^{-}CD57^{low}$ | 514 | 831 | 119 | 308 |
| 7 | Naïve | $CD45RO^{-}CCR7^{+}CD27^{hi}$ $Eomes^{-}T\text{-}bet^{-}$ $CD127^{+}CD70^{-}CD57^{low}$ | 449 | 702 | 128 | 143 |
| 1 | Naïve | $CD45RO^{-}CCR7^{+}CD27^{hi}$ $Eomes^{low}T\text{-}bet^{-}$ $CD127^{+}CD70^{low}CD57^{low}$ | 1235 | 1068 | 183 | 336 |
| 13 | Naïve | $CD45RO^{-}CCR7^{+}CD27^{+}$ $Eomes^{low}T\text{-}bet^{-}$ $CD127^{-}CD70^{low}CD57^{low}$ | 57 | 383 | 66 | 31 |
| 2 | CM/TM | $CD45RO^{+}CCR7^{+}CD27^{+}$ $Eomes^{+}$ | 198 | 375 | 1082 | 624 |
| 6 | CM/TM | $CD45RO^{+}CCR7^{+}CD27^{+}$ $Eomes^{-}$ | 230 | 271 | 700 | 415 |
| 12 | CM/TM | $CD45RO^{+}CCR7^{+}CD27^{-}$ $Eomes^{-}$ | 29 | 125 | 443 | 89 |
| 3 | EM | $CD45RO^{+}CCR7^{-}CD70^{low}CD57^{+}$ | 140 | 101 | 762 | 1044 |
| 8 | EM | $CD45RO^{+}CCR7^{-}CD70^{-}CD57^{+}$ | 117 | 149 | 489 | 513 |
| 10 | EM | $CD45RO^{+}CCR7^{-}CD70^{low}CD57^{hi}$ | 578 | 204 | 269 | 193 |
| 14 | Eff | $CD45RO^{-}CCR7^{-}CD27^{-}$ $Eomes^{+}T\text{-}bet^{+}$ | 18 | 111 | 100 | 243 |
| 11 | Eff | $CD45RO^{-}CCR7^{-}CD27^{+}$ $Eomes^{hi}T\text{-}bet^{low}$ | 216 | 119 | 191 | 223 |
| 9 | Eff | $CD45RO^{-}CCR7^{-}CD27^{-}$ $Eomes^{hi}T\text{-}bet^{+}$ | 283 | 89 | 347 | 533 |

**Table S4.** Detailed information on the percentage of CD4+ T-cells expressed IFNγ, TNF, CD107a and Granzyme B, before (NC) and after stimulation with HCMV pp65 (CMV) and Staphylococcal enterotoxin B super antigen (SEB) on proband (PR), homozygous sibling (HS), a heterozygous relatives (HR) and a healthy control (HC).

| CD4+ T-cells functional data | | | | | | | | | | | | | | | | |
|---|---|---|---|---|---|---|---|---|---|---|---|---|---|---|---|---|
| **Sample** | **Antigen** | **107+Grz+ IFN+TNF+** | **107+Grz+ IFN+TNF-** | **107+Grz+ IFN-NF+** | **107+Grz+IFN-TNF-** | **107+Grz- IFN+TNF+** | **107+Grz- IFN+TNF-** | **107+Grz- IFN- TNF+** | **107+Grz- IFN- TNF-** | **107-Grz+ IFN+TNF+** | **107-Grz+ IFN+TNF-** | **107-Grz+ IFN- TNF+** | **107-Grz- IFN+TNF+** | **107-Grz- IFN+TNF-** | **107-Grz- IFN-TNF+** | **Total response*** |
| PR | NC | 0.026 | 0.026 | 0.026 | 0.033 | 0.026 | 0.026 | 0.026 | 0.101 | 0.026 | 0.026 | 0.044 | 0.026 | 0.076 | 0.040 | ref |
| PR | CMV | 0.026 | 0.029 | 0.026 | 0.028 | 0.029 | 0.032 | 0.026 | 0.026 | 0.032 | 0.035 | 0.027 | 0.041 | 0.029 | 0.068 | 0.117 |
| PR | SEB | 0.041 | 0.026 | 0.032 | 0.026 | 0.103 | 0.035 | 0.121 | 0.081 | 0.326 | 0.176 | 0.063 | 1.066 | 0.486 | 11.312 | 13.556 |
| HS | NC | 0.026 | 0.026 | 0.026 | 0.039 | 0.027 | 0.028 | 0.026 | 0.206 | 0.026 | 0.028 | 0.028 | 0.026 | 0.038 | 0.044 | ref |
| HS | CMV | 0.026 | 0.026 | 0.026 | 0.028 | 0.026 | 0.026 | 0.026 | 0.026 | 0.026 | 0.028 | 0.028 | 0.030 | 0.046 | 0.037 | 0.067 |
| HS | SEB | 0.026 | 0.026 | 0.027 | 0.024 | 0.028 | 0.027 | 0.032 | 0.026 | 0.030 | 0.028 | 0.029 | 0.276 | 0.364 | 1.548 | 2.153 |
| HR | NC | 0.026 | 0.028 | 0.026 | 0.036 | 0.026 | 0.026 | 0.026 | 0.146 | 0.026 | 0.032 | 0.052 | 0.028 | 0.056 | 0.078 | ref |
| HR | CMV | 0.026 | 0.026 | 0.026 | 0.026 | 0.028 | 0.026 | 0.026 | 0.036 | 0.030 | 0.026 | 0.026 | 0.044 | 0.048 | 0.026 | 0.083 |
| HR | SEB | 0.077 | 0.026 | 0.034 | 0.036 | 0.456 | 0.041 | 0.136 | 0.146 | 0.276 | 0.089 | 0.076 | 3.844 | 1.856 | 10.874 | 17.629 |
| HC | NC | 0.026 | 0.026 | 0.026 | 0.055 | 0.026 | 0.026 | 0.026 | 0.166 | 0.026 | 0.047 | 0.043 | 0.030 | 0.065 | 0.065 | ref |
| HC | CMV | 0.032 | 0.026 | 0.028 | 0.012 | 0.026 | 0.026 | 0.026 | 0.026 | 0.032 | 0.026 | 0.026 | 0.041 | 0.033 | 0.021 | 0.044 |
| HC | SEB | 0.586 | 0.031 | 0.026 | 0.000 | 1.996 | 0.083 | 0.206 | 0.096 | 0.786 | 0.105 | 0.149 | 2.432 | 0.677 | 17.587 | 24.422 |

*A total T cell response was here measured by percentage of IFNγ producing cells in total CD4+ T cells after background reduction and comparing to the unstimulated condition (reference level, ref).

Grz: Granzyme B, IFN: IFNγ, 107: CD107a

**Table S5.** Detailed information on the percentage of CD8$^{+}$ T-cells expressed IFNγ, TNF, CD107a and Granzyme B, before (NC) and after stimulation with HCMV pp65 (CMV) and Staphylococcal enterotoxin B super antigen (SEB) on proband (PR), homozygous sibling (HS), a heterozygous relatives (HR) and a healthy control (HC).

| CD8$^{+}$ T-cells functional data | | | | | | | | | | | | | | | | |
|---|---|---|---|---|---|---|---|---|---|---|---|---|---|---|---|---|
| **Sample** | **Antigen** | **107+Grz+ IFN+TNF+** | **107+Grz+ IFN+TNF-** | **107+Grz+ IFN-NF+** | **107+Grz+ IFN-NF-** | **107+Grz- IFN+TNF+** | **107+Grz- IFN+TNF-** | **107+Grz- IFN- TNF+** | **107+Grz- IFN- TNF-** | **107-Grz+ IFN+TNF+** | **107-Grz+ IFN+TNF-** | **107-Grz+ IFN- TNF+** | **107-Grz- IFN+TNF+** | **107-Grz- IFN+TNF-** | **107-Grz- IFN-TNF+** | **Total response*** |
| PR | NC | 0.080 | 0.084 | 0.080 | 0.105 | 0.080 | 0.084 | 0.080 | 0.200 | 0.080 | 0.126 | 0.163 | 0.080 | 0.092 | 0.088 | ref |
| PR | CMV | 0.091 | 0.080 | 0.080 | 0.091 | 0.102 | 0.145 | 0.080 | 0.080 | 0.084 | 0.080 | 0.117 | 0.080 | 0.093 | 0.083 | 0.245 |
| PR | SEB | 0.320 | 0.144 | 0.080 | 0.102 | 0.720 | 0.616 | 0.112 | 0.770 | 0.530 | 0.194 | 0.127 | 0.220 | 0.488 | 0.232 | 3.614 |
| HS | NC | 0.080 | 0.080 | 0.080 | 0.108 | 0.080 | 0.084 | 0.080 | 0.260 | 0.087 | 0.104 | 0.084 | 0.080 | 0.087 | 0.080 | ref |
| HS | CMV | 0.087 | 0.091 | 0.080 | 0.105 | 0.080 | 0.084 | 0.080 | 0.080 | 0.077 | 0.084 | 0.151 | 0.080 | 0.091 | 0.087 | 0.216 |
| HS | SEB | 0.117 | 0.117 | 0.084 | 0.101 | 0.430 | 0.277 | 0.101 | 0.170 | 0.263 | 0.176 | 0.163 | 0.340 | 0.573 | 0.158 | 2.029 |
| HR | NC | 0.080 | 0.080 | 0.080 | 0.175 | 0.080 | 0.080 | 0.080 | 0.220 | 0.080 | 0.106 | 0.240 | 0.080 | 0.089 | 0.097 | ref |
| HR | CMV | 0.089 | 0.080 | 0.080 | 0.080 | 0.145 | 0.172 | 0.080 | 0.080 | 0.089 | 0.100 | 0.140 | 0.089 | 0.108 | 0.080 | 0.373 |
| HR | SEB | 2.630 | 0.410 | 0.091 | 0.275 | 2.900 | 1.610 | 0.124 | 1.110 | 8.480 | 3.134 | 0.040 | 1.390 | 2.441 | 0.873 | 24.468 |
| HC | NC | 0.080 | 0.087 | 0.080 | 0.270 | 0.080 | 0.087 | 0.080 | 0.180 | 0.102 | 0.138 | 0.240 | 0.080 | 0.087 | 0.087 | ref |
| HC | CMV | 0.200 | 0.173 | 0.080 | 0.080 | 0.230 | 0.183 | 0.080 | 0.080 | 0.218 | 0.242 | 0.000 | 0.080 | 0.095 | 0.095 | 0.795 |
| HC | SEB | 2.030 | 1.083 | 0.168 | 1.790 | 2.330 | 1.643 | 0.150 | 1.270 | 1.948 | 2.052 | 0.190 | 0.680 | 1.533 | 0.433 | 16.259 |

*A total T cell response was here measured by percentage of IFNγ producing cells in total CD8$^{+}$ T cells after background reduction and comparing to the unstimulated condition (reference level, ref).

Grz: Granzyme B, IFN: IFNγ, 107: CD107a
