# Supplementary material for: Specific Immune Response and Cytokine Production in CD70 Deficiency
Source: Front Pediatr. 2021 Apr 30;9:615724. doi: 10.3389/fped.2021.615724 (PMC8120026; doi:10.3389/fped.2021.615724)

**Figure S1.** *In vitro* B cell proliferation assay before (non-stimulated) and after stimulation with T-dependent (IL-10+CD40L) cytokines in the proband, the homozygous sibling, a heterozygous relative and healthy control.


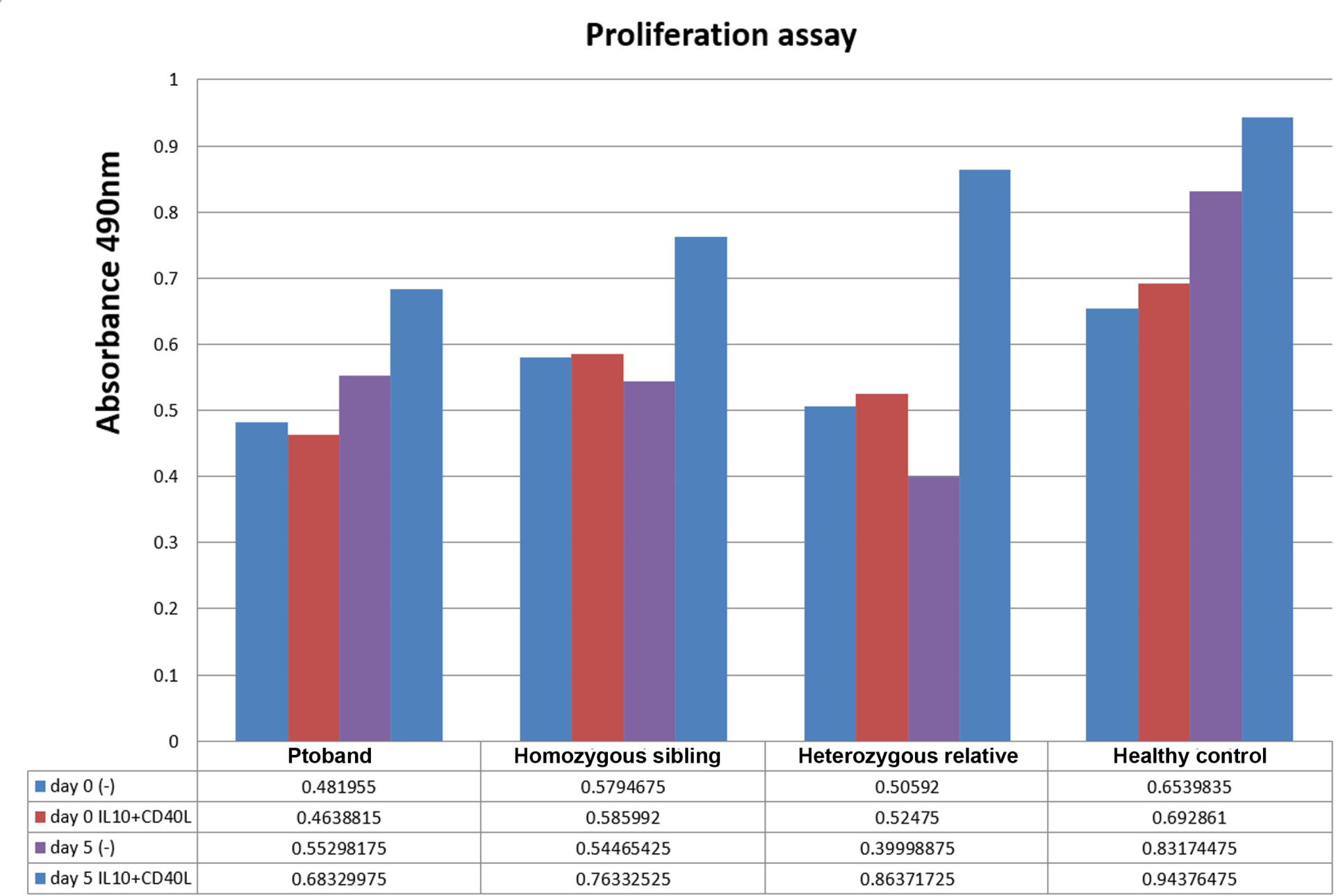

Supplement: Supplementary file 2 [file Figure_1.docx]
